# Fetal Third-Trimester Functional Cardiovascular Abnormalities and Neonatal Elevated Bilirubin Level

**DOI:** 10.3390/jcm12186021

**Published:** 2023-09-17

**Authors:** Maria Respondek-Liberska, Oskar Sylwestrzak, Julia Murlewska, Łucja Biały, Michał Krekora, Monika Tadros-Zins, Ewa Gulczyńska, Iwona Strzelecka

**Affiliations:** 1 Department of Fetal Malformations Diagnosis and Prevention, Medical University of Łódź, 90-419 Łódź, Poland; 2 Department of Prenatal Cardiology, Polish Mother’s Memorial Hospital Research Institute, 93-338 Łódź, Poland; 3 Department of Obstetrics and Gynecology, Polish Mother’s Memorial Hospital Research Institute, 93-338 Łódź, Poland; 4 Students’ Prenatal Cardiology Scientific Group, Medical University of Łódź, 92-213 Łódź, Poland; 5 Department of Neonatology, Intensive Therapy and Neonatal Pathology, Polish Mother’s Memorial Hospital Research Institute, 93-338 Łódź, Poland

**Keywords:** prenatal cardiology, normal heart, tricuspid regurgitation, bilirubin

## Abstract

Background: The aim of the study was to analyze the neonatal outcomes of fetuses with diagnosed functional cardiovascular abnormalities, also considering the connection with neonatal hyperbilirubinemia. Materials and Methods: It was an observational study of 100 neonates who had fetal echocardiography examinations in the third trimester (mean gestational age during the last echocardiography was 34 ± 3 weeks and mean birth weight was 3550 g). There were two groups: A: normal heart anatomy + no functional anomalies; group B: normal heart anatomy + functional abnormalities. Hyperbilirubinemia was defined as a bilirubin level of >10 mg%. Results: In group A, there were 72 cases and only 5 cases despite having normal heart anatomy and normal heart study that presented additional problems. In group B (28 cases), the prenatal functional findings included tricuspid regurgitation (TR) (15 cases, 53%), pericardial effusion (4 cases, 14%), myocardial hypertrophy (4 cases, 14%), cardiomegaly (2 cases, 7%), abnormal bidirectional blood flow across the foramen ovale (3 cases, 11%), aneurysm of atrial septum (2 case, 7%), abnormal E/A ratio for mitral and tricuspid valve (1 case, 3%), bright spot (3 case, 11%), abnormal Doppler flow in ductus arteriosus compared to aortic arch (difference >60 cm/s) (1 case, 3%), supraventricular tachycardia (SVT) (1 case), and mitral regurgitation (1 case, 3%). In group A (*n* = 72 cases), bilirubin levels of >10 mg/dL were observed in 8% of newborns. In group B (*n* = 28), bilirubin levels of > 10 m/dL were observed in 46% of cases and TR was present in group B in 53% of cases (15/28 cases). The difference between group A and B in terms of elevated bilirubin levels was statistically significant (*p* < 0.001). Conclusions: In the case of fetal normal heart anatomy and normal heart study, a good neonatal outcome may be expected. When fetal cardiovascular functional abnormalities in normal heart anatomy are detected, with special attention paid to tricuspid valve regurgitation, a neonatal elevated bilirubin level (mean 11 mg/dL, range 10–15 mg/dL) may be expected.

## 1. Introduction

For more than 40 years, fetal echocardiography has been performed routinely to screen for fetal cardiac anomalies, arrhythmias, and extracardiac malformations. Nevertheless, fetal echocardiography is not only used to detect congenital heart defects (CHD), but also to monitor fetuses, especially those with different pathologies such as functional cardiovascular abnormalities, such as pericardial effusion and valvular insufficiencies, etc. [1]. Many efforts have been made to predict early neonatal outcomes [2,3], but no other method seems to be as detailed as fetal echocardiography. Fetal echocardiography has a special aim depending on the stage of pregnancy. Third-trimester fetal echocardiography, performed close to the delivery term, provides a lot of information regarding the neonatal cardiovascular condition and this subject has been studied widely in case of fetal CHD [4,5,6,7,8,9,10]. However, when normal heart anatomy is found, commonly asked questions include: does fetal echocardiography provide any additional value to obstetricians or neonatologists in the case of normal fetal heart anatomy? The literature regarding fetal functional cardiovascular abnormalities in the third trimester of pregnancy is sparse, especially describing postnatal outcome. The aim of the study was to analyze the neonatal outcomes of fetuses with diagnosed functional cardiovascular abnormalities, also considering the connection with neonatal elevated bilirubin level.

## 2. Materials and Methods

This observational study was based on 100 consecutive newborns who underwent third-trimester prenatal ultrasound and echocardiography and “Normal Heart Anatomy” (NHA), without extracardiac malformation (anomalies that require any type of neonatal surgery). The database of our referral unit for prenatal cardiology was analyzed, from July 2022 to February 2023. 

The 100 cases were placed into two groups:

Group A: normal fetal heart anatomy + normal heart study + neonatal outcome;
Group B: normal fetal heart anatomy + functional abnormalities + neonatal outcome;

### 2.1. Defining of Functional Abnormalities

Prenatal functional anomalies are, for example: tricuspid valve regurgitation (TR), defined as a systolic flow of >1.5 m/s and duration of >80 ms [11], with its presence both in color Doppler and spectral Doppler; septal hypertrophy, defined as a septum thickness of >4.5 mm regardless of gestational age [12]; pericardial effusion (PE), defined in the case of >3 mm; and cardiomegaly, defined as a heart area/chest area of >0.45 [13].

The ultrasound machine was a Voluson E 10 with a 3–6 MHz abdominal probe.

After birth, the bilirubin level was measured via a laboratory test from the neonatal blood.

### 2.2. Statistical Evaluation

For the statistical analysis, the Statistica 13.1 and Excel 2007 programs were used. Continuous variables were expressed as means, and qualitative variables as numbers (%). The normality of the distribution of the continuous variables was checked using the Shapiro–Wilk test. The qualitative variables were compared between groups using the chi-square test (*p* < 0.05). The continuous variables were compared between groups using the U Mann–Whitney test (*p* < 0.05).

The data of the patients were deidentified as this was an observational study. The way of medical management was not changed, and the authors focused on the interpretation of additional collected data; thus, new approval from the Ethical Committee was not necessary.

## 3. Results

The mean maternal age was 32 ± 5 years (range, 22–46) and the mean gestational age during last echocardiography was 34 ± 3 weeks (based on last menstrual period). All fetuses were appropriate for gestational age (AGA) and the mean birth weight was 3550 g (there were no premature or small for gestational age fetuses).

### 3.1. Group A

Group A: there were 72 cases (72%) and 5 cases (Table 1) that required additional explanation (despite having a normal heart anatomy and normal heart study in fetal echocardiography):

Case 1 (“D”) had a heart murmur and the neonatal echocardiography revealed a 2 mm muscular ventricular septal defect (VSD), but it did not require any treatment and the VSD closed spontaneously 4 weeks later.

Case 2 (“N”) underwent fetal echocardiography four times due to Turner syndrome, diagnosed by a Non-invasive Fetal Trisomy Test (NIFTY), and had a normal heart anatomy and no prenatal functional anomalies.

Case 3 (“L”) was diagnosed with Down Syndrome during the first trimester by NIFTY and had fetal echocardiography three times, without any evidence of functional anomalies.

Case 4 (“W”) had no prenatal abnormalities detected in fetal echocardiography, presented with mild transient respiratory problems and dysmorphic features as a newborn, suggestive of Fetal Alcohol Syndrome.

Case 5 (“C”) was treated with Vancyclovir for maternal cytomegalovirus infection and had no other problems during fetal life and no functional heart or extracardiac abnormalities.

### 3.2. Group B

**Figure 1:**
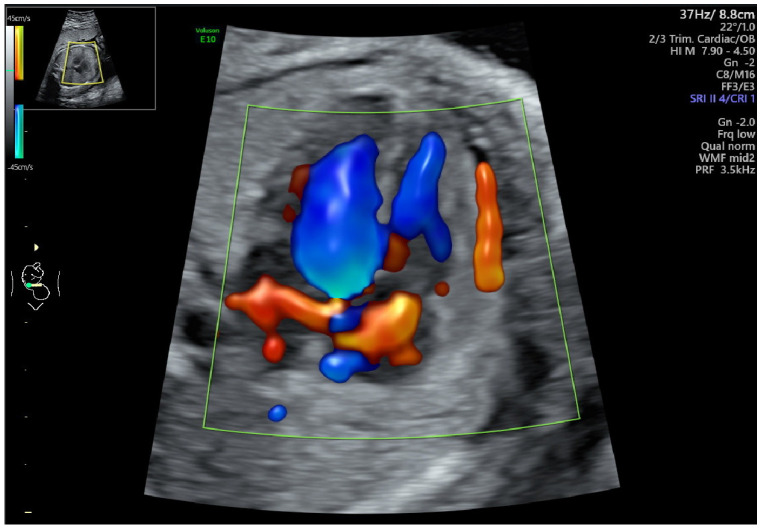
Fetal pericardial effusion.

**Figure 2:**
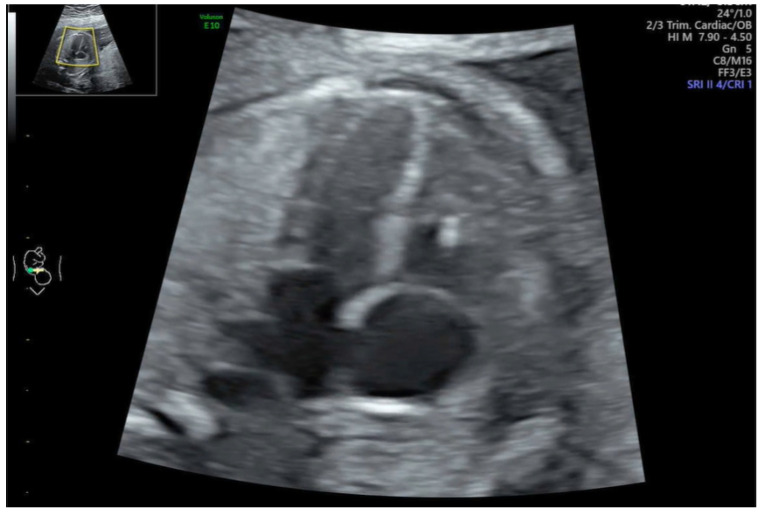
Fetal bright spot in the right ventricle.

Group B: N = 28 cases (28%) (Table 2): the prenatal functional findings included TR (15 cases, 53%), PE (4 cases, 14%) (Figure 1), myocardial hypertrophy (4 cases, 14%), cardiomegaly (2 cases, 7%), abnormal bidirectional blood flow across the foramen ovale (3 cases, 11%), aneurysm of the atrial septum (2 case, 7%), abnormal E/A ratio for mitral and tricuspid valve (1 case, 3%), bright spot (BS) (Figure 2) (3 case, 11%), abnormal Doppler flow in ductus arteriosus (DA) compared to aortic arch (difference >60 cm/s) (1 case, 3%), supraventricular tachycardia (SVT) (1 case), and mitral regurgitation (1 case, 3%). In Case 1 with prenatal TR, the newborn required resuscitation in the delivery room, but was doing well later and was discharged on day 4. In eight cases, despite prenatal functional anomalies, the newborns’ conditions were good: alive and well (A & W).

### 3.3. Bilirubin Level

**Figure 3:**
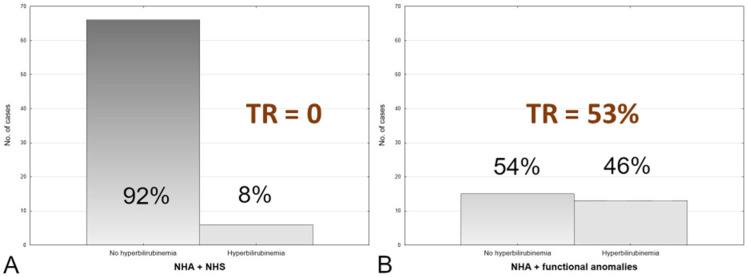
Elevated bilirubin level distribution in the Group (**A**) (72 cases) and Group (**B**) (28 cases).

In group A, the prevalence of a bilirubin level of >10 mg/dL was 8%, and in group B, it was 46% (*p* < 0.00005) (Table 3). The prevalence of TR in group A was 0%, and in group B, it was 53% (mean 15%). A chi-square test was performed for elevated bilirubin level (>10 mg/dL) between group A and group B *p* < 0.0005 (Figure 3).

## 4. Discussion

Based on our observations, fetal echocardiography in the third trimester of pregnancy, showing a normal heart anatomy, normal heart study, and no extracardiac malformations nor anomalies, was a very good prognostic predictor of a positive neonatal outcome. None of the group A newborns had low Apgar scores, required treatment in the intensive care unit, required surgery, or died.

The echocardiographic examinations were performed in high-risk pregnancies in tertiary centers with different indications for such detailed exams (maternal age, positive family history, previous child with heart defect, in vitro fertilization (IVF) pregnancy, maternal chronic disease, and abnormal screening test in first trimester). This is why, during the study period, we had only 100 cases with a normal heart anatomy. Moreover, this may explain the high percentage of observed cardiovascular abnormalities. There are many differences in fetal and neonatal echocardiography. In our center, in the case of a normal result for fetal echocardiography, neonatal echocardiography was not routinely performed, but in the case of an abnormal fetal result, neonatal echocardiography was provided to ensure and improve the diagnosis. Thus, both fetal and neonatal echocardiography stand to supplement each other in order to provide a maximally detailed diagnosis.

In the group of fetuses with a normal heart anatomy and functional heart anomalies, the most common functional abnormality was TR. In the total group, this was 15%, which is higher than reported previously. The tricuspid valve usually has three cusps and each is supported by chordae tendineae. An injury of the tricuspid leaflets or chordate tendinae may result in TR. According to the first publication of functional TR by Respondek [11], its prevalence in normal heart anatomy was 6.8% and 6.2% by Gembruch [14], 4.5% by Clerici [15], 6.7% by Zhou from China [16], and 5.2% by Wiechec [17]. The higher prevalence of TR in this study could be attributed to the current “post-COVID-19 era” (eight pregnant women from Table 2 and Table 4 had vaccinations during their pregnancy and another five had COVID-19 symptoms during pregnancy), but this is just a working hypothesis, and no additional details were taken for analysis.

According to the Donofrio group, mild/trivial TR is a frequent finding after 34 weeks of gestation and, in isolated cases, can be explained as normal and a result of the physiologic alterations that occur late in the third trimester [18]. Similar conclusions were formulated by Messing and co-workers [19]. The high prevalence of mild TR suggests that this phenomenon may reflect a normal physiological finding during early fetal development, which decreases in prevalence and severity over the course of pregnancy.

However, an interesting speculation was presented by Zhou [16], that the presence of fetal isolated TR may alter the flow dynamics in the right atrium, resulting in the delayed closure of the interatrial septum, leading to a patent foramen ovale or atrial septal defects.

That said, to explore this possibility, one needs to conduct much a longer follow-up beyond 1 month of postnatal life. For our analysis, we had only routine data from the hospital Clininet program and analyzed only early postnatal outcomes.

The pathomechanisms of fetal functional TR can be explained by a systolic overload of the right ventricle, a diastolic overload of the right ventricle, or a decreased myocardium contractility [20,21]. Furthermore, a common hemodynamic pathophysiology is right heart dysfunction with moderate to severe tricuspid regurgitation in the setting of a low perfusion state. Right heart dysfunction in infants with CHD can result in severely conjugated bilirubin, likely as a consequence of venous congestion, and can be used as an indirect marker of right heart dynamics [22].

As none of our cases required pharmacological treatment due to premature labor, no steroids, and no pain killers in medical anamnesis, there were no signs of ventricular overload, and the only possible pathomechanism seemed to be slight myocardium impairment, which was not noticed by routine fetal echocardiography in our center. This included Tei RV, Tei LV, and RV- and LV-shortening fractions, and TAPSe and MAPSe but not a HQ evaluation, which probably could be more sensitive for this purpose, with a delineation of not only ventricles, but also the area of the atria, which plays a significant hemodynamic role during fetal life [23].

Other authors did underline the meaning of fetal TR during the first trimester of pregnancy and correlation between fetal TR and congenital heart defects or genetic syndromes [24,25,26,27], but we focused on normal heart anatomy and no extracardiac malformations during the third trimester.

An interesting and new observation from our study was the relatively frequent presence of elevated bilirubin levels (however, these ranges were still considered as physiological jaundice) in the newborns with functional abnormalities: 12 out of 24 cases (50%) (regardless of the duration time for newborn hospitalization). In a group of 72 newborns with a prenatally normal heart anatomy and no functional abnormalities (group A), the bilirubin was elevated (defined as >10 mg%) in six (8%). The mean bilirubin level in this group was 11.3 ± 1 mg/dL and, in group B, it was 13.5 ± 2 mg/dL, and there was no statistical difference (*p* = 0.168). However, the level of bilirubin in the neonates who, as fetuses, had functional anomalies, compared to the group with no functional abnormalities, was significant (*p* < 0.00005).

The explanation of fetal functional tricuspid valve regurgitation and neonatal elevated bilirubin level could be related to a primary temporary impairment of fetal liver function (due fetal viral infection or even without infection), which may change the heart preload. On the other hand, fetal TR could raise the blood pressure in the right atrium, inferior vena cava, and then in the hepatic vessels. That could be the cause of later impaired bilirubin conjugation. After birth and a breathing room air afterload change, there is more blood going into the neonatal lungs, and heart function is going to improve. However, a former prenatal increase in preload would be seen in neonates as a temporarily elevated bilirubin level. This is a hypothesis and further studies would be required in the future to check such a possibility.

To support the “infection” hypothesis, we will need to make a quotation of the case report from our center, when the longitudinal fetal and neonate observation did confirm that persistent TR preceded neonatal pneumonia and a bilirubin level of 13 mg/dL on day 6 [28].

The problem of transient hemodynamic changes with atrio-ventricular regurgitations has been described in newborns after asphyxia [29], so probably some discomfort of the fetuses may be expressed as transient functional tricuspid regurgitation.

An interesting additional observation from our study was that detailed ultrasound and echocardiographic examinations did not show any anomalies in the selected cases of Down Syndrome, Turner Syndrome, Fetal Alcohol Syndrome, and maternal CMV infection, even with known prenatal diagnoses after the first-trimester positive screening.

An interesting continuation of the study would be an analysis regarding the long-term follow-up of fetuses with a normal heart anatomy and cardiovascular abnormalities. As presented by Barker DJ, the origin of some cardiovascular pathologies may start in utero life and detected fetal cardiovascular abnormalities could potentially be connected to adult diseases, but this theory needs more profound life-long observational studies [30]. For further studies, established normal ranges for ultrasound and echocardiographic parameters should be used to unify results [31,32].

## 5. Conclusions

In the case of fetal normal heart anatomy and normal heart study, there is no need for cardiosurgical or invasive management. All the neonates were stable with no urgent cardiovascular deterioration. When fetal cardiovascular functional abnormalities in the normal heart anatomy are detected, with special attention paid to TR, a neonatal elevated bilirubin level (mean 11 mg/dL, range 10–15 mg/dL) may be expected.

## Figures and Tables

**Table 1:** Selected cases from Group A: normal heart anatomy, no prenatal functional anomalies, and some additional problems (but none of the had bilirubin level of >10 mg%).

Case (Initial)	Prenatal Genetic Dgn	Fetal ECHO	Postnatal Dgn	Comments	Hospital Stay (Days)
1	None	Normal anatomy, no functional anomalies	2 mm VSD	During fetal and postnatal life no additional functional anomalies	3
2	Turner S (NIFTY 1 trim)	Normal anatomy, no functional anomalies	Normal heart	Low hairline, widely positioned nipples, puffy feet	3
3	Down S (NIFTY 1 trim)	Normal anatomy, no functional anomalies	Normal heart	Dysmorphic face	4
4	None	Normal anatomy, no functional anomalies	Normal heart	Fetal Alcohol Syndrome	30
5	Maternal CMV	Normal anatomy, no functional anomalies	Normal heart	Alive and well	19

Abbreviations: CMV—cytomegalovirus; NIFTY, non-invasive fetal trisomy test; and VSD, ventricular septal defect.

**Table 2:** Normal heart anatomy + prenatal functional abnormalities (*n* = 28 cases).

Case	Prenatal Diagnosis	Postnatal Diagnosis	Delivery	Birth Weight	Apgar	Bilirubin mg/Dl and Other Data	Days in Hospital
1	NHA + TR.	NHA	V	3300	6	Neonatal resuscitation	4
2	NHA + FO spinnaker	NHA + FO	Cs	3530	10		4
3	NHA + abnormal E/A ratio for MV and TV, SGA	NHA	Cs	2380	10		4
4	NHA + TR	NHA + FO	Cs	3120	10		4
5	NHA + TR	NHA	Cs	2680	9		4
6	NHA + PAC	NHA	V	3280	10		4
7	NHA + TR + PE	NHA + FO	V	3390	10		4
8	NHA + TR + AFI + Placentitis + hydrops testis	NHA	V	3620	10		4
9	NHA + TR + BS+ PE	NHA	V	2570	10		4
10	NHA + TR + bowel distended, Pyelectasis, AFI	NHA Polydactyly	V	2790	8	16	4
11	NHA + Myocardial hypertrophy	NHA	V	3660	10	Green amniotic fluid 14.5	4
12	NHA + TR + PE + FO	NHA	V	3260	10	15	4
13	NHA + PE + FO	NHA		3230	10	15	4
14	NHA+ TR + KMP	NHA	Cs	2780	10	Green amniotic fluid	4
15	BS, FO spinnaker	NHA	CS	3180	10	Positive blood culture, the next one negative	5
16	NHA + DA/Ao + placentitis	NHA	V	3230	10	10	5
17	NHA + FO + DV velocity > 100 cm/s + placentitis + oligohydramnion	NHA	V	4030	10	15	5
18	NHA + cardiomegaly	Normal anatomy, no functional anomalies	CS	3140	10	12.5 Green amniotic fluid	5
19	Cardiomegaly, TR, Ao arch	NHA + TR	V	2900	10	TR, 10	6
20	TR	NHA	CS	3900	9	11.45	6
21	TR, MR, AFI, placentitis	Normal anatomy	V	2310	9	GBS (+)	6
22	Cardiomegaly, BS	Normal anatomy	CS	3220	8	Positive blood culture, the next one negative	6
23	TR	Normal anatomy	CS	3870	8	CRP 14 Sat 87%	6
24	TR	Normal anatomy Cryptorchismus	CS	3670	10	GBS (+) 12	8
25	NHA + TR + KMP + placentitis	Normal anatomy	V	3550	10	16.1	8
26	NHA + UMB	Normal anatomy	CS	2470	8	CRP 10 conjunctivitis	11
27	LGA, Hypertrophy	Normal anatomy	CS	3400	10	Green amniotic fluid	16
28	SVT	SVT, X-ray	CS	2400	9	Atelectasis, Streptococcus cristatus, Staphylococcus hominis	21
				3173 ± 474	9.7 ± 0.9	Mean bilirubin 12 ± 2 mg/dL	6 ± 3

**Table 3:** Analysis of bilirubin level (>10 mg/dL) in study group.

	Group A: Normal Heart Anatomy & No Functional Anomalies	Group B: Normal Heart Anatomy + Functional Anomalies	Total
Total number of cases (fetuses)	72	28	100
Number of cases with bilirubin ≥10 mg/dL	6	13	19
% of cases with elevated bilirubin level	8%	46%	19%

Chi-square test for elevated bilirubin level (≥10 mg/dL) between group A and group B *p* < 0.0005.

**Table 4:** Analysis of prevalence of fetal tricuspid valve regurgitation TR (*n* = 100).

	Group A: Normal Heart Anatomy	Group B: Normal Heart Anatomy + Prenatal TR	Total
Total number of cases (fetuses)	72	28	100
Number of cases with TR	0	15	15
% of cases with TR	0%	53%	15%

Chi-square test for elevated bilirubin level (≥10 mg/dL) between group A and group B *p* < 0.0005.

## Data Availability

Data available after reasonable request.
